# The Anticonvulsant Enaminone E139 Attenuates Paclitaxel-Induced Neuropathic Pain in Rodents

**DOI:** 10.1155/2013/240508

**Published:** 2013-12-08

**Authors:** Dhandapani Thangamani, Ivan Ogheneochuko Edafiogho, Willias Masocha

**Affiliations:** ^1^Department of Pharmacology and Therapeutics, Faculty of Pharmacy, Kuwait University, P.O. Box 24923, 13110 Safat, Kuwait; ^2^Department of Pharmaceutical Sciences, School of Pharmacy, University of Saint Joseph, Hartford, CT 06103, USA

## Abstract

The enaminone methyl 4-(4′-bromophenyl)aminocyclohex-3-en-6-methyl-2-oxo-1-oate (E139) has anticonvulsant activities. It has been reported to have a better safety profile than some anticonvulsant drugs. Since some anticonvulsant drugs are used in the management of neuropathic pain, we evaluated the effects of E139 in rodent models of acute pain and paclitaxel-induced neuropathic pain. The reaction latency to thermal stimuli (hot-plate test) of BALB/c mice was recorded before and after intraperitoneal treatment with paclitaxel (2 mg/kg, i.p. for 5 consecutive days), and after treatment with E139 (0.1–40 mg/kg), amitriptyline (10 mg/kg), and gabapentin (10 and 30 mg/kg). Mechanical allodynia in paclitaxel-treated Sprague Dawley (SD) rats was measured using a dynamic plantar aesthesiometer before and after treatment with E139 (10 and 20 mg/kg) or its vehicle for four consecutive days from day 7 after first administration of paclitaxel (16 mg/kg on two alternate days). Administration of E139 (10–40 mg/kg) produced antinociceptive activity against thermal nociception in naïve mice. Treatment with E139, amitriptyline, or gabapentin reduced paclitaxel-induced thermal hyperalgesia. E139 reduced paclitaxel-induced mechanical allodynia, with the effects lasting longer (24 h) after repetitive dosing. Our results indicate that E139 has antinociceptive activity and attenuates paclitaxel-induced neuropathic pain in rodents.

## 1. Introduction

Paclitaxel (taxol), a taxane with mitotic spindle inhibitory activity, is a key component of regimens used in the treatment of various solid tumors including ovarian, breast, and lung cancer. Its use, however, is negatively affected by the development of dose-limiting painful peripheral neuropathy [1]. Neurotoxicity is considered the major nonhematologic side effect of paclitaxel [2]. Paclitaxel causes acute pain syndrome, impairs sensory fibers, and produces peripheral neuropathy that present as a chronic painful neuropathic syndrome in some of the patients with symptoms that include hyperalgesia, allodynia, and spontaneous sensations such as burning, shooting, numbness, spasm, and prickling [3, 4].

Currently, there are no proven effective drugs for the prevention or treatment of paclitaxel-induced neuropathic pain (PINP) or chemotherapy-induced peripheral neuropathy (CIPN) in general. Tricyclic antidepressants such as amitriptyline and anticonvulsants such as gabapentin have been used for symptomatic treatment of CIPN. Unfortunately, these drugs often have no significant benefit, provide only partial relief, or have dose limiting side effects [5, 6]. Therefore, new alternative drugs are needed. Enaminones, which are enamines of *β*-dicarbonyl compounds, have been reported to have anticonvulsant effects in *in vitro* and *in vivo* models with minimal side effects [7–9]. The antinociceptive potential of enaminones has not yet been evaluated. The aim of this study was to evaluate the antinociceptive effects of the enaminone methyl 4-(4′-bromophenyl)aminocyclohex-3-en-6-methyl-2-oxo-1-oate (E139) in naïve mice and in mice and rat models of PINP that manifest mechanical allodynia or thermal hyperalgesia [10, 11]. E139 has been reported to suppress tetrodotoxin- (TTX-) sensitive sodium channels and enhance extracellular levels of GABA [8, 12]. Sodium channels have been implicated in the pathogenesis of PINP [13] and the alteration of the GABAergic system plays a role in chronic pain syndromes [14]; thus, E139 might have antinociceptive effects against PINP.

## 2. Materials and Methods

### 2.1. Animals

The animals used in this study were female BALB/c mice (8 to 12 weeks old; 20–30 g, *n* = 176) and male Sprague Dawley rats (8 to 12 weeks old; 200–300 g, *n* = 67) supplied by the Animal Resources Centre at the Health Sciences Center, Kuwait University, Kuwait. Animals were kept in temperature controlled (24 ± 1°C) rooms with food and water *ad libitum*. All experiments were performed during the same period of the day (8:00 AM to 4:00 PM) to exclude diurnal variations in pharmacological effects. The animals were handled in compliance with European Communities Council Directive 86/609 for the care of laboratory animals and ethical guidelines for research in experimental pain with conscious animals [15]. All procedures were approved by the Ethical Committee for the use of Laboratory Animals in Teaching and in Research, Health Sciences Centre, Kuwait University.

### 2.2. Administration of Paclitaxel to Induce Neuropathic Pain

**Figure 1:**
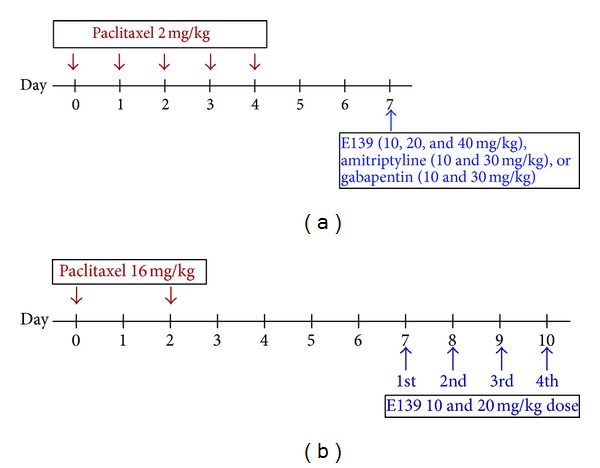
Drug administration schedule for (a) mice and (b) rats. The arrows indicate the days when the drugs were intraperitoneally administered.

Paclitaxel (Tocris, Bristol, UK) was dissolved in a solution made up of 50% Cremophor EL and 50% absolute ethanol to a concentration of 6 mg/mL and stored at −20°C, for a maximum of 14 days. For treatment of mice the 6 mg/mL paclitaxel solution was then diluted in normal saline (NaCl 0.9%), to a final concentration of 0.2 mg/mL just before administration. The vehicle for paclitaxel was diluted at the time of injection with normal saline in the same proportion as the paclitaxel solution. Paclitaxel 2 mg/kg or its vehicle were administered to mice intraperitoneally (i.p.), in a volume of 10 mL/kg, once per day for 5 consecutive days; the cumulative dose of paclitaxel was 10 mg/kg (the paclitaxel administration schedule for mice is depicted in Figure 1(a), illustration adapted from Hidaka et al. [16]). This treatment regimen has been reported to produce painful neuropathy and thermal hyperalgesia in mice [11, 13]. Paclitaxel 16 mg/kg or its vehicle, without dilution with NaCl 0.9%, were administered to rats intraperitoneally (i.p.) as previously described [10], in a volume of 2.667 mL/kg, on two alternate days; the cumulative dose was 32 mg/kg (the paclitaxel administration schedule for rats is depicted in Figure 1(b)).

### 2.3. Drug Administration

E139 was synthesized in-house [7], dissolved in peanut oil, and administered i.p. to mice at a volume of 5 mL/kg body mass and to rats at a volume of 2.5 mL/kg body mass. E139 (0.1–40 mg/kg) was administered to naïve mice and to paclitaxel treated mice at 7 days after first administration of paclitaxel, when mice had developed thermal hyperalgesia as previously described [11]. Amitriptyline (Sigma-Aldrich, St Louis, MO, USA; 10 and 30 mg/kg) and gabapentin (Sigma-Aldrich, St Louis, MO, USA; 10 and 30 mg/kg) were dissolved in normal saline and administered to paclitaxel treated mice at 7 days after first administration of paclitaxel. The E139, amitriptyline, and gabapentin administration schedule for mice is depicted in Figure 1(a). E139 (10 and 20 mg/kg) was administered to rats for four consecutive days from day 7 after first administration of paclitaxel, when rats had developed mechanical allodynia (the E139 administration schedule for rats is depicted in Figure 1(b)).

### 2.4. Assessment of Thermal Nociception

Reaction latencies to hot-plate test were measured before (baseline latency), at day 7 after first injection of paclitaxel and at various times starting at 30 minutes after E139, amitriptyline, or gabapentin treatment. Briefly, mice were individually placed on a hot plate (Panlab SL, Barcelona, Spain) with the temperature adjusted to 55 ± 1°C. The time to the first sign of nociception, paw licking, flinching, or jump response to avoid the heat was recorded and the animal immediately was removed from the hot plate. A cut-off period of 20 seconds was maintained to avoid damage to the paws. Percent change in reaction latency was calculated as follows: [(response latency after drug treatment − pretreatment baseline latency)/pretreatment baseline latency] × 100.

### 2.5. Assessment of Mechanical Allodynia

Mechanical allodynia in rats was measured using the dynamic plantar aesthesiometer (Ugo Basile, Italy), as previously described [10, 17] and following the procedures described by the manufacturer. Briefly, rats were left to habituate for about 15 minutes inside plastic enclosures on top of a perforated platform before starting a microprocessor which was programmed to automatically lift a metal filament that exerted a linearly increasing force (2.5 g/s with cut-off time of 20 s) on the hind paw. A stop signal was automatically attained, either when the animal removed the paw or at the cut-off force of 50 g. Withdrawal thresholds in response to the mechanical stimulus was automatically recorded in grams. The hind paws were tested at least 3 times with a 3–5 minute interval. Percent change in withdrawal force was calculated as follows: [(withdrawal force after drug treatment − pretreatment baseline withdrawal force)/pretreatment baseline withdrawal force] × 100.

### 2.6. Statistical Analyses

Statistical analyses were performed using Student's *t*-test, one-way analysis of variance (ANOVA) followed by Dunnett's multiple comparison test, Grubbs' test, Kruskal-Wallis test, or two-way repeated measures ANOVA followed by Bonferroni posttests. The differences were considered significant at *P* < 0.05.

## 3. Results

### 3.1. Effects of Treatment with E139 in a Hot-Plate Test in Naïve BALB/c Mice

**Figure 2:**
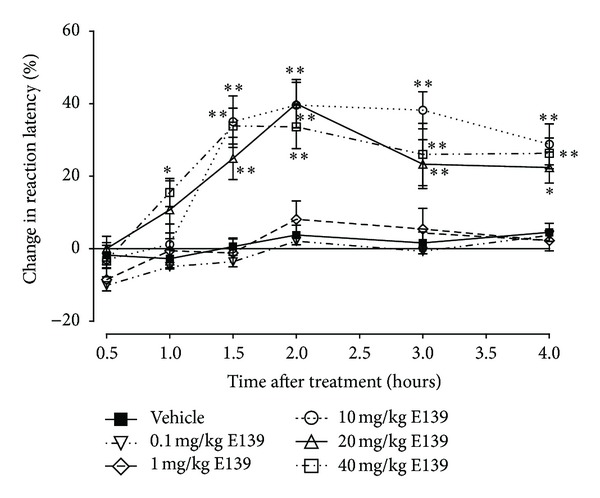
Time course of the percentage change in reaction latency time for various doses of E139 (0.1–40 mg/kg) in a hot-plate test in naïve BALB/c mice. Each point represents the mean ± S.E.M of values obtained from 10 animals. *P < 0.05 and **P < 0.01 compared to drug vehicle at the same time point after treatment (two-way repeated measures ANOVA followed by Bonferroni test).

The intraperitoneal administration of vehicle or lower doses of E139 (0.1 and 1 mg/kg) did not change the reaction latency to thermal stimulation (*P* > 0.05), whereas higher doses (10, 20, and 40 mg/kg) produced significant percentage increase in reaction latency compared to vehicle-treated mice (*P* < 0.05; Figure 2). There was a significant interaction between treatment and time after treatment for E139 doses of 10 mg/kg (*F*_5,90_ = 12.43, *P* < 0.0001), 20 mg/kg (*F*_5,90_ = 4.72, *P* = 0.0007), and 40 mg/kg (*F*_5,90_ = 6.40, *P* < 0.0001). The effect of E139 was time dependent; from the time of administration it rose to a peak between 1.5 and 3 h and was still significantly high at 4 h.

### 3.2. Effects of E139, Amitriptyline, and Gabapentin on Paclitaxel-Induced Thermal Hyperalgesia

**Figure 3:**
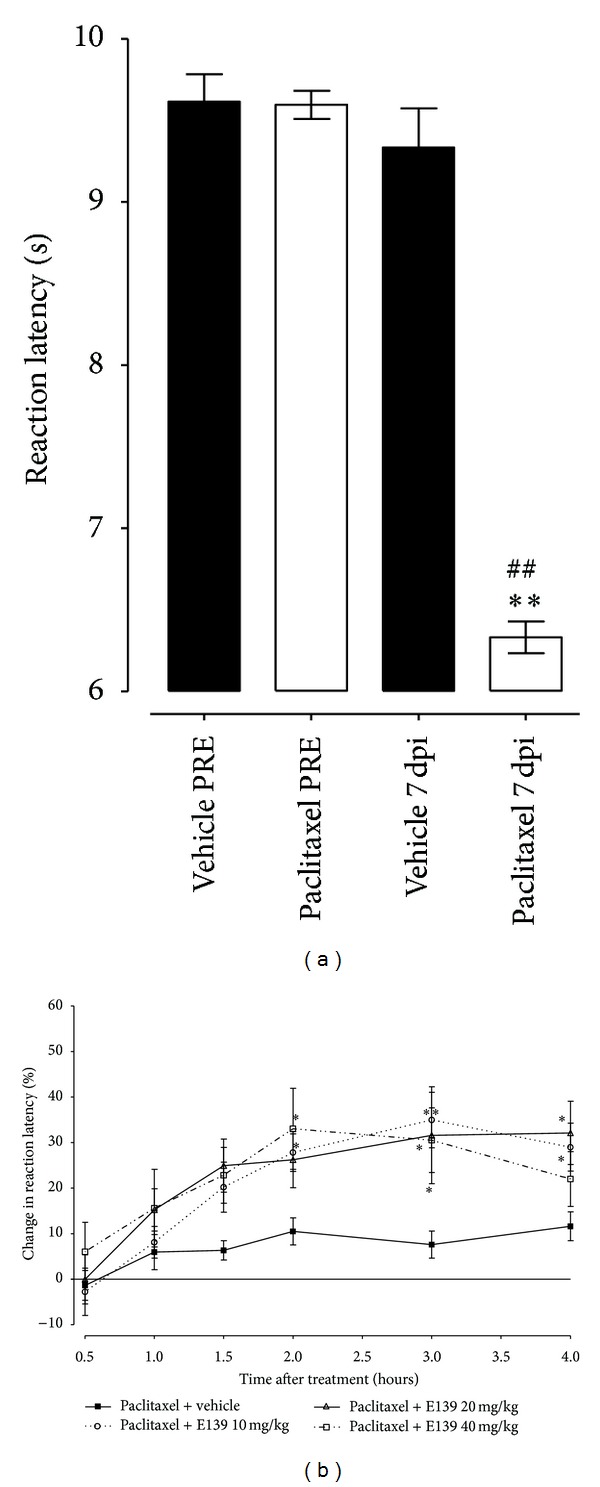
Effect of treatment with E139 on BALB/c mice with paclitaxel-induced thermal hyperalgesia in a hot-plate test. (a) Thermal hyperalgesia in BALB/c mice at day 7 after first inoculation of paclitaxel (administered at 2 mg/kg, i.p. for 5 consecutive days). Each point represents the mean ± S.E.M of values obtained from 22 vehicle-treated and 94 paclitaxel-treated animals. **P < 0.01 compared to drug vehicle at the same day after treatment and ^##^ *P* < 0.01 compared to pretreatment (PRE) values (Student's *t*-test). (b) Percentage change in reaction latency times from baseline values (taken at day 7 after first administration of paclitaxel) at different times after treatment with E139 (10–40 mg/kg) or its vehicle in hot-plate test. Each bar represents the mean ± S.E.M of values obtained from 12 animals. **P < 0.01 compared to drug vehicle at the same time point after treatment (two-way repeated measures ANOVA followed by Bonferroni test).

Paclitaxel produced a significant reduction in response latency time to thermal stimuli (thermal hyperalgesia) on day 7 after first drug administration compared to the baseline latency (pretreatment values) and vehicle-only-treated animals in the hot-plate test (*P* < 0.01; Figure 3(a)), as previously described [11].

The intraperitoneal administration of E139 (10, 20, and 40 mg/kg) produced significant percentage increase in reaction latency in mice with paclitaxel-induced thermal hyperalgesia compared to vehicle-treated animals (*P* < 0.05, Figure 3(b)). There was a significant interaction between treatment and time after treatment for E139 doses of 10 mg/kg (*F*_5,110_ = 5.65, *P* = 0.0001) and 20 mg/kg (*F*_5,110_ = 2.30, *P* = 0.0494) but not 40 mg/kg (*F*_5,110_ = 1.96, *P* = 0.0909). The effect of E139 was time dependent; from the time of administration it produced significant effects from 2 h onwards.

**Figure 4:**
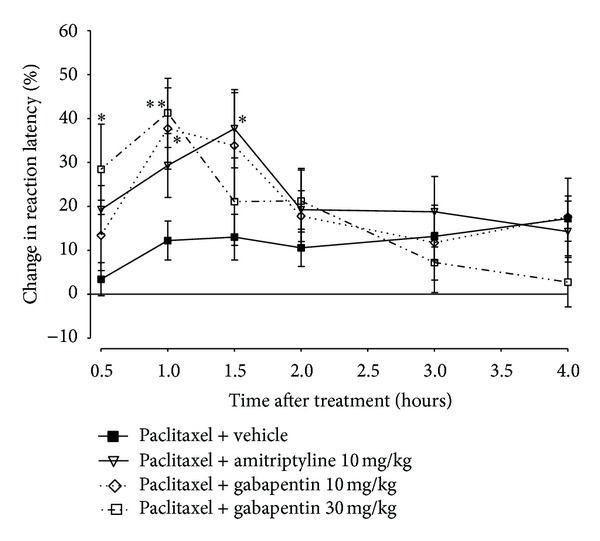
Effect of treatment with amitriptyline and gabapentin on BALB/c mice with paclitaxel-induced thermal hyperalgesia in a hot-plate test. Percentage change in reaction latency times from baseline values (taken at day 7 after first administration of paclitaxel) at different times after treatment with amitriptyline (10 mg/kg), gabapentin (10 and 30 mg/kg), or their vehicle in a hot-plate test. Each bar represents the mean ± S.E.M of values obtained from 8–16 animals. *P < 0.05 compared to drug vehicle at the same time point after treatment (two-way repeated measures ANOVA followed by Bonferroni test).

The intraperitoneal administration of amitriptyline at a dose of 10 mg/kg, which has been shown to reduce thermal hyperalgesia in another model of neuropathic pain [18], also produced significant percentage increase in reaction latency in mice with paclitaxel-induced thermal hyperalgesia compared to vehicle-treated animals (*P* < 0.05, Figure 4). There was a significant interaction between treatment and time after treatment for amitriptyline 10 mg/kg (*F*_5,130_ = 3.45, *P* = 0.0058). The effect of amitriptyline was also time dependent, producing significant effects earlier than E139 at 1.5 h after administration but was also short lived, having no significant effects 2 h after administration. The peak effect of amitriptyline 10 mg/kg at 1.5 h was 37.7 ± 8.9% increase in reaction latency, which was earlier than but similar in magnitude (*P* > 0.05) to that of E139 10 mg/kg (at 3 h and 35.0 ± 6.1% increase in reaction latency). A higher dose of amitriptyline (30 mg/kg) caused sedation and thus was not evaluated in the hot-plate test.

The intraperitoneal administration of gabapentin, which has been shown to reduce paclitaxel-induced thermal hyperalgesia [19], 10 and 30 mg/kg also produced significant percentage increase in reaction latency in mice with paclitaxel-induced thermal hyperalgesia compared to vehicle-treated animals (*P* < 0.05, Figure 4). There was a significant interaction between treatment and time after treatment for gabapentin doses of 10 mg/kg (*F*_5,110_ = 3.72, *P* = 0.0038) and 30 mg/kg (*F*_5,110_ = 9.29, *P* < 0.0001). The effect of gabapentin was also time dependent, producing significant effects earlier than E139 at 0.5 h after administration but was also short lived, having no significant effects 1.5 h after administration. The peak effects of gabapentin 10 and 30 mg/kg at 1 h were 37.7 ± 9.3% and 41.3 ± 7.9% increase in reaction latency, respectively, which were earlier than but similar in magnitude (*P* > 0.05) to that of E139 10 mg/kg (at 3 h and 35.0 ± 6.1% increase in reaction latency).

### 3.3. Effects of E139 on Paclitaxel-Induced Mechanical Allodynia

**Figure 5:**
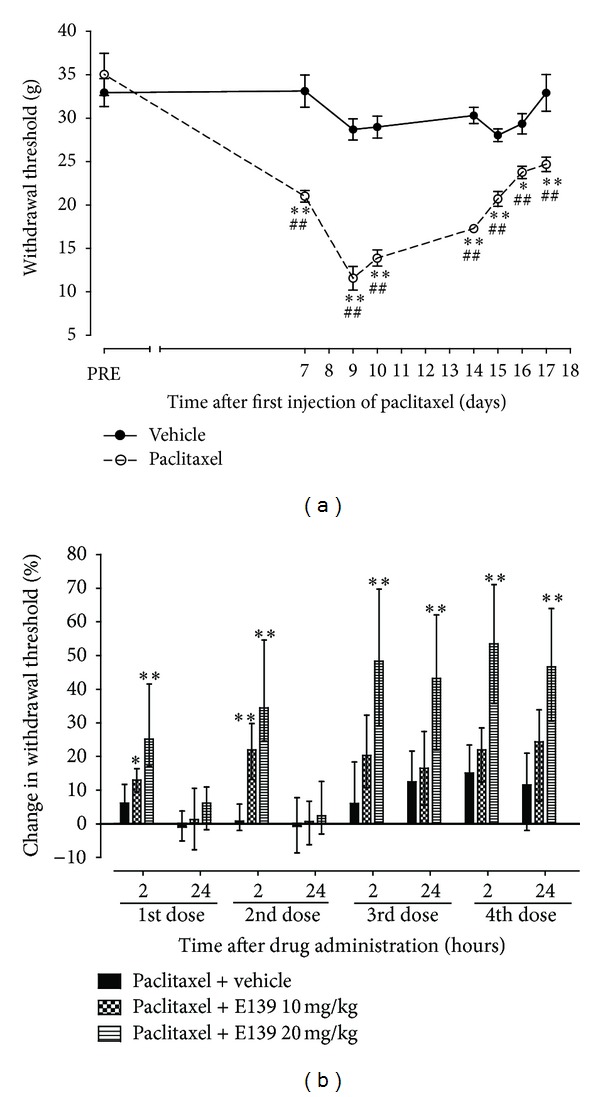
Effect of treatment with E139 on paclitaxel-induced mechanical allodynia in Sprague Dawley rats. (a) Time course of the withdrawal threshold to the dynamic plantar aesthesiometer after administration of paclitaxel 16 mg/kg or its vehicle on two alternate days. Each point represents the mean ± S.E.M of values obtained from 9 animals. *P < 0.05 and **P < 0.01 compared to drug vehicle at the same day after treatment (two-way repeated measures ANOVA followed by Bonferroni test) and ^##^ *P* < 0.01 compared to pretreatment (PRE) values (one-way ANOVA followed by Dunnett's multiple comparison test). (b) Percentage change in withdrawal threshold from baseline values (taken at day 7 after first administration of paclitaxel) at 2 and 24 h after each treatment with E139 (10 and 20 mg/kg) or its vehicle using a dynamic plantar aesthesiometer. E139 was administered in 4 daily doses. Each bar represents the median values with interquartile range of values obtained from 16 to 17 animals. *P < 0.05 and **P < 0.01 compared to drug vehicle at the same time point after treatment (Kruskal-Wallis test).

Paclitaxel produced a significant reduction in response withdrawal threshold to mechanical stimuli (mechanical allodynia), as previously described [10], from 7 to 17 days after first drug administration compared to the baseline latency and vehicle-only-treated animals measured using the dynamic plantar aesthesiometer (*P* < 0.05; Figure 5(a)). There was a significant interaction between treatment with paclitaxel and time after treatment (*F*_7,128_ = 11.08, *P* < 0.0001). The nadir of the reduction of the withdrawal threshold was on day 9, where paclitaxel treated rats had a withdrawal threshold of 11.6 ± 1.4 g compared to 35.0 ± 2.4 g before paclitaxel treatment, almost 70% reduction in withdrawal threshold.

Rats with paclitaxel induced mechanical allodynia were treated with E139 (10 and 20 mg/kg) or its vehicle daily for four consecutive days from day 7 after first administration of paclitaxel. One of the rats with paclitaxel-induced mechanical allodynia treated with vehicle produced values which were significant outliers (*P* < 0.05, Grubbs' test performed using http://graphpad.com/quickcalcs/Grubbs1.cfm). The values obtained from this animal were presented together with those obtained from the rest of the other animals in this group as median values with interquartile range and the data in this set was analyzed using a nonparametric statistical test, Kruskal-Wallis test.

The intraperitoneal administration of E139 10 mg/kg significantly increased the withdrawal threshold in rats with paclitaxel-induced mechanical allodynia compared to vehicle-treated animals only for the first two doses and at 2 h (*P* < 0.05) but not at 24 h after drug administration (*P* > 0.05; Figure 5(b)). On the other hand the intraperitoneal administration of a higher dose of E139 20 mg/kg significantly increased the withdrawal threshold in rats with paclitaxel-induced mechanical allodynia compared to vehicle-treated animals at 2 h (*P* < 0.05) after drug administration (*P* < 0.05; Figure 5(b)) throughout the duration of the treatment (four doses). The effects of E139 20 mg/kg lasted for 24 h (*P* < 0.05) starting from the third administration of the drug.

## 4. Discussion

This study shows for the first time that an anticonvulsant enaminone E139 has antinociceptive activities in naïve mice and attenuates paclitaxel-induced thermal hyperalgesia and mechanical allodynia in rodents.

E139 has been shown to have anticonvulsant activities *in vivo* in animals with minimal side effects. It was found to have a median toxic dose (TD_50_) of 270 mg/kg in rats [20], which was far above the highest dose we used, 40 mg/kg, and found to have antinociceptive activities. E139 did not display any motor impairment in mice at a dose of 50 mg/kg [20]. In rats E139 neither produced neurotoxicity at a dose of 110 mg/kg nor did it elicit ataxia [20]. On the other hand, amitriptyline an antidepressant drug which has been used for symptomatic management of CIPN [5, 21] had sedative effects in paclitaxel-treated BALB/c mice at a dose of 30 mg/kg, the same dose has been observed to induce somnolence in rats [22]; thus we had to limit the dose we evaluated for antinociceptive activity to 10 mg/kg. Amitriptyline is known to have dose-limiting sedative effects [23, 24] and has also been reported to cause ataxia [25]. Somnolence, dizziness, and ataxia are also dose-limiting side effects of gabapentin [25–27] an anticonvulsant drug that has also been used for symptomatic management of CIPN [21, 28].

E139 produced antinociceptive effects against thermal nociception in naïve mice similar to what has been observed with amitriptyline or gabapentin [29–32]. We observed antinociceptive effects of E139, amitriptyline, and gabapentin in mice with paclitaxel-induced thermal hyperalgesia. We did not find studies describing the effects of amitriptyline against paclitaxel-induced thermal hyperalgesia, but the drug was found to have antinociceptive effects in other models of neuropathic pain [18, 33]. On the other hand, gabapentin has been shown to have antinociceptive effects in mice with paclitaxel-induced thermal hyperalgesia [19].

Mechanical allodynia is a well-known feature in rats treated with paclitaxel [34, 35] and the effects of both amitriptyline and gabapentin have been studied on symptomatic relief of established paclitaxel-induced mechanical allodynia [36, 37]. E139 attenuated paclitaxel-induced mechanical allodynia from the first dose, however, repeated dosing was necessary for the effects of the drug to be long lasting (i.e., up to 24 h after treatment). This pattern to some extent is similar to what has been observed with both gabapentin and amitriptyline [36, 37]. However, the immediate effects of E139 were observed earlier than either compound; that is, the first dose of E139 had some antiallodynic effect whilst amitriptyline and gabapentin did not [36, 37]. One of the differences between these studies was that we examined the effects of E139 at 2 and 24 h, whereas Xiao et al. examined the effects of amitriptyline and gabapentin at 1 and 24 h [36, 37]. E139 and amitriptyline's antiallodynic effects lasted up to 24 h after repetitive dosing whereas that of gabapentin did not [36, 37].

E139 has been reported to block TTX-sensitive sodium currents and enhance extracellular levels of GABA possibly via activation of *α*2-adrenoceptors in* in vitro *models of epilepsy [8, 12, 38, 39]. These activities of E139 are possibly responsible for its antinociceptive effects in rodent models of neuropathic pain. Systemic administration of low doses of TTX has been shown to have antinociceptive/antiallodynic activity in mice with paclitaxel-induced thermal hyperalgesia and mechanical allodynia [13]. Amitriptyline blocks sodium channels [40–42] and this has been suggested to be one of its possible mechanisms of activity against paclitaxel-induced neuropathic pain [37]. The antinociceptive effects of amitriptyline have also been attributed to its action on *α*2-adrenoceptors [43] and this and its effects on other *α*-adrenoceptors are possibly responsible for its effects against neuropathic pain, including that induced by paclitaxel [37, 44, 45]. All these are in addition to its principal effects as a serotonin and noradrenaline reuptake inhibitor. Activation of GABA receptors using baclofen has also been shown to have antiallodynic activities in rats with paclitaxel-induced mechanical allodynia but not as consistently as amitriptyline [37]. Gabapentin, an anticonvulsant drug which acts via binding to *α*2*δ*-1 subunit containing voltage-gated calcium channels also has antinociceptive/antiallodynic effects in rodents with paclitaxel-induced neuropathic pain [19, 36, 37]. Taking into consideration our current findings and literature sources, the activities of E139 against neuropathic pain and possibly the mechanism of actions are more similar to that of amitriptyline than gabapentin. However, E139 is devoid of the sedative effect of amitriptyline.

## 5. Conclusions

In conclusion the data obtained in this study show that the anticonvulsant enaminone E139 has antinociceptive activity, and attenuates paclitaxel-induced neuropathic pain. The antiallodynic activities of E139 are longer lasting after repetitive treatment; thus, repetitive dosing of E139 has potential therapeutic benefits in the management of paclitaxel-induced neuropathic pain.
